# Surgical trauma‐induced CCL18 promotes recruitment of regulatory T cells and colon cancer progression

**DOI:** 10.1002/jcp.27245

**Published:** 2018-09-14

**Authors:** Zhirong Sun, Chunchun Du, Pingbo Xu, Changhong Miao

**Affiliations:** ^1^ Department of Anesthesiology Fudan University Shanghai Cancer Center Shanghai China; ^2^ Department of Oncology Shanghai Medical College, Fudan University Shanghai China

**Keywords:** C‐C motif chemokine ligand 18 (CCL18), colon cancer, surgical, tregs and immunosuppressive environment

## Abstract

**Background:** Surgical stress has been suggested to facilitate colon cancer growth and metastasis. However, the precise mechanisms by which surgical trauma promotes colon cancer progression remain poorly understood.

**Methods:** To unravel the mechanisms underlying surgery‐induced colon cancer progression, a syngenic transplantation tumor model was established with CT26 cells, and the effect of laparotomy on tumor progression was investigated. Especially, the expression of several chemokines was assessed, and their roles in recruiting CD4+ CD25+ regulatory T cells (Tregs) after surgery were analyzed.

**Results:** Tregs population was significantly increased in the tumor tissue and peripheral blood of tumor‐bearing mice after laparotomy. C‐C motif chemokine ligand 18 (CCL18) expression was significantly upregulated after laparotomy in tumor tissue and the peritoneal cavity of tumor‐bearing mice, and it was positively correlated with the recruitment of Tregs. Functionally, CCL18 knockdown significantly reduces tumor growth and angiogenesis compared with control. Through analysis of Tregs, we found an upregulated proportion of Tregs in tumor tissue, peritoneal cavity, and peripheral blood after laparotomy, but this enhancement was blocked after CCL18 knockdown. In patients with colon cancer, a higher Tregs proportion is positively correlated to more advanced clinical TNM stages and shorter survival. Furthermore, a positive correlation was found between the serum CCL18 level and the Treg proportion in clinical samples.

**Conclusion:** Surgical trauma contributes to colon cancer progression by increasing CCL18 expression and hence promotes Treg recruitment, which leads to an immunosuppressive environment.

## 1 INTRODUCTION

Colorectal cancer (CRC) is a common type of malignant tumor with more than one million new cases diagnosed every year worldwide (Siegel, Miller, & Jemal, 2015). The disease‐specific lethality of CRC has decreased gradually in the past decades with the application of surgical methods, and, more recently, it has been demonstrated that surgical trauma is likely correlated with CRC‐associated tumor development and metastasis (Li et al., 2013; Tai et al., 2013). However, the precise reasons behind the fact that surgical trauma is responsible for inducing the progression of cancer cells, especially triggering the immune system, have not been fully understood.

Chemokines are characterized as the key regulators of the immune system, which are associated with the recruitment of leukocytes to inflammatory sites as well as secondary lymphoid organs (Singh, Lillard, & Singh, 2011). It is also frequently reported that chemokines play a key role in malignant transformation and tumor progression (Payne & Cornelius, 2002). A group of chemokines (CXCL9, CXCL10, and CXCL11) were identified as potential biomarkers for personalized prognosis of colon cancer (Kistner, Doll, Holtorf, Nitsche, & Janssen, 2017; Lin et al., 2007). High intratumoral expression of these chemokines is indicative of good prognosis (Kistner et al., 2017). Kapur et al. (2016) reported that C‐C motif chemokine receptor 6 (CCR6) level is higher in colon cancer cases compared with normal adjacent tissue, and CCR6 might be a potential therapeutic target as well as a biomarker in addition to nodal status for predicting therapeutic options.

C‐C motif chemokine ligand 18 (CCL18), a member of the CC chemokine family, was reportedly expressed in monocytes, macrophages, and immature dendritic cells and associated with the progression of malignant tumors (Meng et al., 2014). Increased CCL18 production has been found in various tumor tissues in the presence of several malignancies. A previous study suggested that the maturation and recruitment of killer cells, including lymphocytes and dendritic cells, are suppressed by the highly expressed CCL18 protein. Also, it interrupts the immunocompetence of killer cells (Schutyser, Richmond, & Van Damme, 2005; Zhang et al., 2013). In a previous pathway analysis of a genome‐wide association study, CCL18 was suspected to be an influencing factor for pancreatic cancer development via the Th1/Th2 immune response (Li et al., 2012). Hence, CCL18 shows the potential of being a crucial regulator in cancer progression and metastasis.

Regulatory T cells (Tregs), characterized by expression of CD25 and forkhead box P3 (Foxp3), are a group of CD4+ T cells (Zou, 2006). Tregs are reported to play a key role in maintaining the immune tolerance by suppressing the activation and proliferation functions of immune cells (Sakaguchi, Yamaguchi, Nomura, & Ono, 2008), whereas, in favor of tumor progression, Tregs can also suppress anticancer immune response (Sakaguchi, Miyara, Costantino, & Hafler, 2010). Accumulating evidence has suggested that Tregs can express chemokine receptors, which have influence on their migration through binding to specific ligands (Zhu & Paul, 2008).

In the current study, we investigated whether surgical trauma contributes to the progression of colon cancer by upregulating CCL18 and recruiting Tregs. This study was conducted to analyze the possible role of CCL18 in promoting tumor progression after laparotomy, where Tregs was recruited in the presence of CCL18.

## 2 MATERIALS AND METHODS

### 2.1 Colon cancer samples

The study was approved by the Institutional Review Boards of the Fudan University Shanghai Cancer Center and an informed consent was signed by all patients. Thirty‐nine patients (Age, 69.5 ± 8.2; Gender, 23 Males and 16 Females; Race, Han Chinese) with colon cancer were selected from March 24, 2013 to December 30, 2017, and all patients were diagnosed by two pathologists. The study methodologies were approved and set by the local ethics committee and the standard of the Declaration of Helsinki, respectively.

### 2.2 Mice surgery model

Six‐week‐old female BALB/c mice were purchased from Chinese Academy of Sciences (Shanghai, China) and bred under sterile condition. Mice were grouped randomly into control and surgical groups. CT26 colon cancer cells (2–5 × 10^6^ cells in 100 μl 1640 medium) were injected subcutaneously into the right flanks of BALB/c mice, and the tumor condition in mice was monitored daily. The laparotomy was then performed 4 days after cell injection to mimic the effects of surgery. The abdominal surgery trauma was performed as previously described in our unpublished manuscript. Briefly, the mice were anesthetized followed by an incision on the abdominal wall and a defect on the omentum, and the wound was then sutured. The control group was treated with shaving, disinfection, and an epidermal injury. The volumes of tumor were determined every 7 days, and after 15 days, some mice were killed with removal of the tumors. Lv/shCCL18 was obtained from Genepharm (Shanghai) to specifically inhibit CCL18 expression, and its effectiveness was verified in our laboratory. The tumor volume was calculated as follow: Volume = length × width^2^ × 0.5.

### 2.3 Quantitative real‐time polymerase chain reaction

Total RNA was extracted from tumor tissues or cells using Trizol reagent following the manufacturer's instruction. The Oligo dT primer and M‐MLV Reverse Transcriptase Kit were used for reverse transcription. The SYBR Green Supermix was used for quantitative real‐time polymerase chain reaction amplification on an Applied Biosystems 7300 real‐time PCR system (Applied Biosystems, CA). β‐Actin was used as reference for mRNA. All samples were examined in triplicates. The primers sequences are shown in Supporting Information Table S1.

### 2.4 Immunohistochemistry

Tumor tissues were fixed in 10% formalin and embedded in paraffin followed by cutting into 5‐μm sections and mounted on slides. After antigen repairing, the sections were incubated overnight with the primary antibodies as follow: 1:50 anti‐CD31 (ab28364; Abcam, CA), 1:100 anti‐CCL18 (22303‐1‐AP; Proteintech, IL). After washing three times, the samples were then incubated with the secondary antibody (HRP‐labeled goat anti‐rabbit, 1: 200, CST). The expressions of CD31 and CCL18 were observed using DAB staining.

### 2.5 Enzyme‐linked immunosorbent assay

Enzyme‐linked immunosorbent assay (ELISA) was performed to determine the protein level of CCL18 according to the manufacturer's instructions (R&D systems, Minneapolis, MI). The mice ascites were collected and stored at −80°C. Centrifugation was performed at 3,000 rpm for 15 min, and supernatants were collected and diluted, followed by ELISA.

### 2.6 Flow cytometry analysis

Flow cytometry analysis was performed to analyze the content of Tregs. Cells were collected from peripheral blood followed by washing and incubation for 10 min at 4°C with buffer. Antibodies CD4 and Foxp3 (BD Biosciences, San Diego, CA) were used to select Tregs. The cells were then washed twice and fixed with 0.4% formaldehyde in buffer. The Guava InCyte system (Life Science) was used for analysis.

### 2.7 Statistical analysis

All data were shown as mean ± standard error and analyzed using Student's *t* test or one‐way analysis of variance. Differences were considered statistically significant when the *p* value < 0.05.

## 3 RESULTS

### 3.1 Laparotomy promotes tumor growth and angiogenesis in vivo

**Figure 1:**
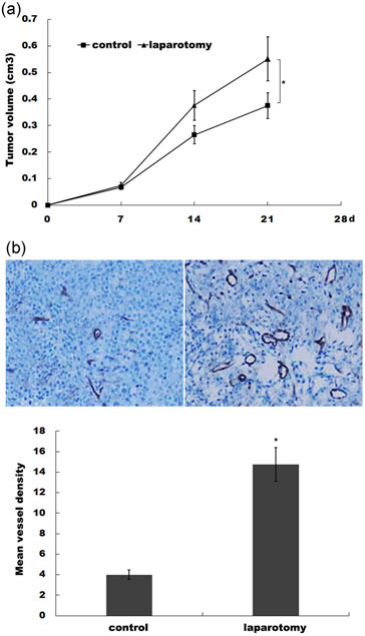
Laparotomy promotes tumor growth and angiogenesis in vivo. (a) CT26 cells were subcutaneously injected into BALB/c and laparotomy was performed 4 days after cell injection. Tumor volume was calculated by the formula length × width^2^ × 0.5, which approximates the volume of an elliptical solid. *p < 0.05. (b) CT26 tumor samples from control or surgically animals were stained for CD31, and the numbers of vessels were quantified after immunohistochemistry (IHC). *p < 0.05 [Color figure can be viewed at wileyonlinelibrary.com]

Emerging studies have reported that surgical stress promotes tumor growth in multiple types of cancer, including colon cancer, ovarian cancer, and liver cancer (Lee et al., 2009; Li et al., 2013; Tohme et al., 2016). To investigate the mechanism underlying the fact that surgical stress facilitates tumor growth and metastasis of colon cancer, a transplantation tumor model was established with colon cancer CT26 cells and the effect of laparotomy on tumor progression was assessed. Two weeks after laparotomy or the sham operation, the tumor volumes of two groups were significantly differentiated. Figure 1a shows that abdominal surgery resulted in a significantly higher tumor volumes compared with control. In addition, we observed a higher vessel density in tumor tissues of the laparotomy group using immunohistochemical staining analysis (Figure 1b).

### 3.2 Laparotomy results in an increase in treg population in tumor tissue and peripheral blood

**Figure 2:**
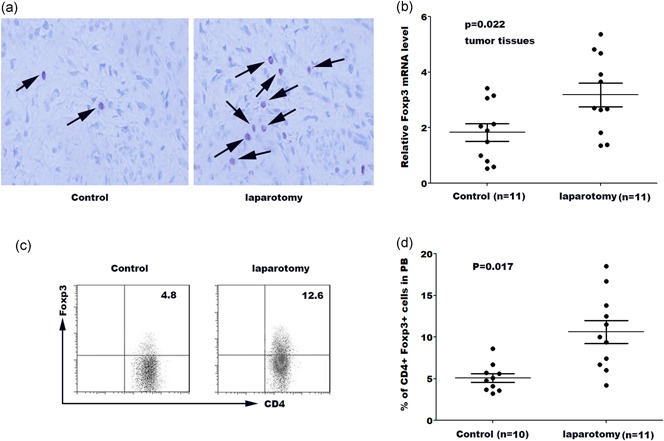
Laparotomy results in an increase in Treg population in tumor tissue and peripheral blood. (a) Double staining for FOXP3 and CD4 in the laparotomy group and the control group, observed under microscopy. (b) qPCR analysis of Foxp3 mRNA level in tumor tissues in laparotomy group and control group. (c) Mice from sham control and surgery were killed at Day 14 and tumor tissues were rinsed with PBS. The lavage fluids were collected and Tregs in the lavage fluids were measured by flow cytometry analysis. (d) Quantitative analysis of the percentage of Tregs in peripheral blood of mice from different groups. FOXP3: forkhead box P3; mRNA: messenger RNA; qPCR: quantitative real‐time polymerase chain reaction [Color figure can be viewed at wileyonlinelibrary.com]

Treg is a critical subpopulation of T cells, which suppress effector T‐cell responses as well as the activity of other immune cells, such as mast cell, dendritic cells, and B cells (Lin et al., 2013; Zhuo et al., 2014). Within the tumor microenvironment, Tregs are coopted by cancer cells to escape immune surveillance (Lv et al., 2014). Elevated proportions of Tregs in tumor microenvironment usually correlated with unfavorable prognosis in several kinds of cancers (Tao et al., 2012). We thus assayed the frequencies of Tregs in cancer tissues after laparotomy. Double staining for FOXP3 and CD4 showed that Foxp3+CD4+ cells were increased in cancer tissues of laparotomy group (Figure 2a). We then assessed the messenger RNA (mRNA) expression levels of Foxp3 in each tumor between the sham control and laparotomy groups. As shown in Figure 2b, the Foxp3 mRNA level was significantly increased after laparotomy. We also assayed the frequencies of Tregs (clarified as a percentage of CD4+FOXP3+ cells/total CD4+ cells) in peripheral blood using flow cytometry. Figure 2c,d showed that Tregs in peripheral blood were markedly increased in mice with laparotomy than controls.

### 3.3 Laparotomy results in an increase in CCL18 expression

**Figure 3:**
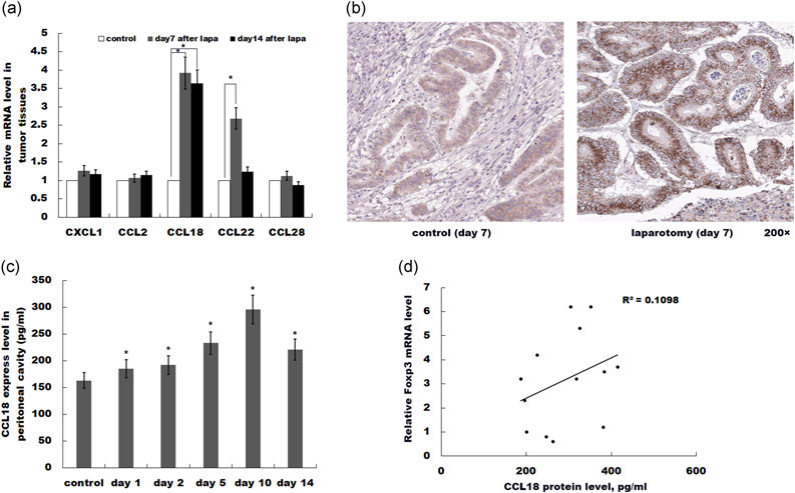
Laparotomy results in an increase in CCL18 expression. (a) qPCR analysis of CXCL1, CCL2, CCL18, CCL22, and CCL28 mRNA level in tumor tissues of sham control or laparotomy. CCL18 mRNA levels were significantly upregulated on Day 7 and 14, while other chemokines were uninfluenced. *n* = 5. *p < 0.05. (b) IHC analysis of CCL18 protein in tumor tissues of sham control or laparotomy at Day 7. (c) ELISA analysis of CCL18 protein expression in the peritoneal cavity. *p < 0.05 versus control. (d) Foxp3 percentage is positively correlated with relative CCL18 expression. *R* ^2^ = 0.1098, *p* = 0.035. CCL18: C‐C motif chemokine ligand 18; ELISA: enzyme‐linked immunosorbent assay; Foxp3: forkhead box P3; IHC: immunohistochemistry [Color figure can be viewed at wileyonlinelibrary.com]

Chemokines play an important role in several pathological processes such as inflammation, immunity diseases, and cancer progression (Kistner et al., 2017; Singh et al., 2011). CXC‐chemokines regulates angiogenesis and immune cells recruitment, which connects cancer cells and the surrounding stroma (Oladipo et al., 2011). We then investigated the functional contribution of chemokines CXCL1, CCL2, CCL18, CCL22, and CCL28, described as Tregs attractants. We assessed the mRNA expression levels of these chemokines between the sham control and laparotomy groups at Day 7 and Day 14. As shown in Figure 3a, only CCL18 mRNA level was significantly upregulated at Day 7 and Day 14 after laparotomy. Immunohistochemistry (IHC) analysis was carried out to assess the CCL18 protein level between the control and laparotomy groups. Figure 3b showed that CCL18 protein level was upregulated after laparotomy compared with control. ELISA assay confirmed that CCL18 protein level in the peritoneal cavity was increased for two weeks after laparotomy (Figure 3c). Furthermore, a positive relationship was found between the Foxp3 level and the CCL18 level in tumor tissues (Figure 3d, *R* ^2^ = 0.1098, *p* = 0.035).

### 3.4 CCL18 promotes the recruitment of Tregs

**Figure 4:**
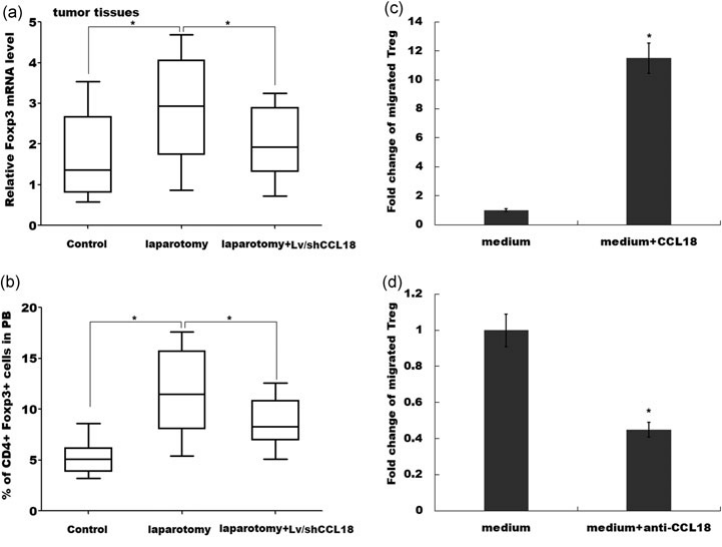
CCL18 promotes the recruitment of Tregs. (a) qPCR analysis of Foxp3 mRNA level in the control group, laparotomy group and laparotomy+Lv/shCCL18 group. *n* = 7. (b) Flow cytometry analysis of Tregs level in peripheral blood of control group, laparotomy group, and laparotomy+Lv/shCCL18 group. *n* = 7. The migration behavior of Tregs isolated from normal mice were examined using transwell migration assays in the absence or presence of CCL18 (c) or CCL18 antibody (d). The number of cell migration was quantified. *p < 0.05. CCL18: C‐C motif chemokine ligand 18; Foxp3: forkhead box P3; mRNA: messenger RNA; qPCR: quantitative real‐time polymerase chain reaction

Tregs are significantly overproduced in tumor tissues after laparotomy. Tregs infiltrate into tumor tissues and modulate the tumor microenvironment to promote tumor growth (Argon, Vardar, Kebat, Erdinç, & Erkan, 2016). Recent studies showed that Tregs were recruited into tumor tissues to promote colon cancer progression after abdominal surgery (Sundstrom et al., 2016). We thus investigated whether upregulated CCL18 promotes the recruitment of Tregs into tumor tissues. Foxp3 mRNA level was significantly increased in tumor tissues after laparotomy, whereas knockdown of CCL18 partially destroyed laparotomy‐induced upregulation of Foxp3 (Figure 4a). As expected, Tregs in peripheral blood were markedly increased in mice with laparotomy, whereas CCL18 knockdown significantly inhibited laparotomy‐induced Tregs accumulation (Figure 4b). To investigate the direct effect of CCL18 on recruiting Tregs, Tregs were freshly isolated from healthy mice and added to the top chambers of Transwell plates. As shown in Figure 4c, CCL18 markedly promoted the migratory activity of the Tregs, whereas downregulation of CCL18 significantly inhibited Tregs migration (Figure 4d).

### 3.5 CCL18 knockdown inhibits tumor growth and angiogenesis

**Figure 5:**
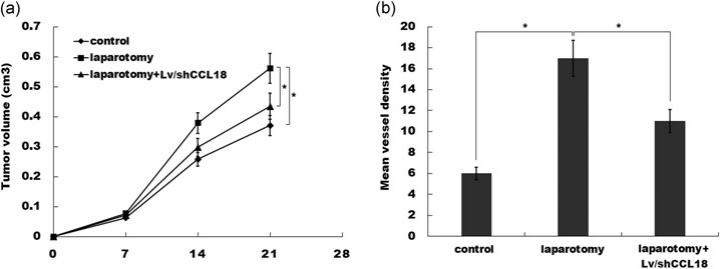
CCL18 knockdown inhibits tumor growth and angiogenesis. (a) CT26 cells and Lv/shCCL18‐treated CT26 cells were subcutaneously injected into BALB/c, and laparotomy was performed 4 days after cell injection. Tumor volume was measured at the indicated time from different groups. *p < 0.05. (b) CT26 tumor samples from control or surgically animals were stained for CD31 and vessel density was calculated by tracing CD31+ vessel. *p < 0.05

Next, we addressed whether aberrant CCL18 expression was a reason for tumor growth and angiogenesis. As expected, abdominal surgery resulted in a significantly higher tumor volumes compared with control, whereas knockdown of CCL18 partially destroyed laparotomy‐induced tumor growth (Figure 5a). Similarly, a higher vessel density was observed in tumor tissues of laparotomy group, whereas knockdown of CCL18 suppressed laparotomy‐induced angiogenesis (Figure 5b).

### 3.6 The Treg percentage is correlated with clinical overall survival rates

**Figure 6:**
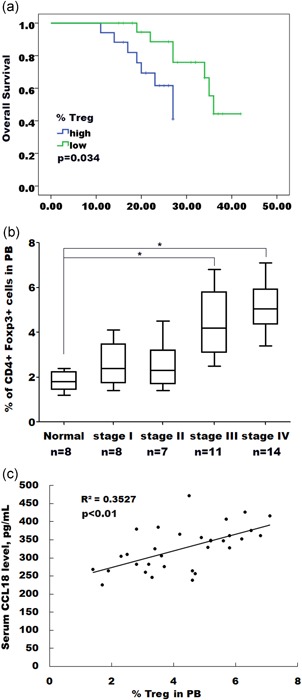
The Treg percentage is correlated with clinical overall survival rates. (a) The overall survival rates of colon cancer patients are negatively correlated to the Tregs percentage. (b) Whole blood drawn from colon cancer patients was analyzed for the presence of Tregs. The results showed a significant correlation of circulating Tregs percentage with tumor stage. *p < 0.05. (c) Tregs percentage is positively correlated with relative CCL18 expression. *R* ^2^ = 0.3527, *p* < 0.01; CCL18: C‐C motif chemokine ligand 18 [Color figure can be viewed at wileyonlinelibrary.com]

The above data strongly implied that CCL18 promotes colon cancer progression in tumor‐bearing mice by regulating the recruitment of Tregs. Then, we analyzed the survival data of the colon cancer patients whose tumor samples had been investigated for Tregs percentage by flow cytometry analysis. For Tregs, the high percentage group showed a shorter survival compared with the low percentage group (Figure 6a). We investigated the correlation of circulating Tregs with colon cancer stage. As shown in Figure 6b, the percentage of circulating Tregs is significantly correlated with colon cancer stage. Furthermore, a positive relationship was found between Tregs percentage and CCL18 expression (Figure 6c; *R* ^2^ = 0.3527, *p* < 0.01). Taken together, these results demonstrated that surgical trauma contributes to progression of colon cancer by upregulating CCL18 expression and hence promoting Tregs recruitment, resulting in an immunosuppressive environment.

## 4 DISCUSSION

The effect of surgical trauma on promoting the tumor progression is commonly recognized by the suppression of host immune function in the presence of surgical trauma, which eventually creates an environment in favor of tumor cell development. Clinical studies discovered that patients with laparoscopy showed a greater number of lymphocyte and TH1 cytokines than those with conventional laparotomy (Evans et al., 2009). A previous study used BALB/c mice to conduct the laparotomy and revealed an increase in the PI3K/AKT‐dependent tumor growth (Coffey, Wang, Bouchier‐Hayes, Cotter, & Redmond, 2006). Meanwhile, increased expressions of EGFR and HER2 were reported to be involved in the enhanced tumor growth and chemoresistance induced by surgical stress from a laparotomy (Amin et al., 2010). In the current study, our results provided direct evidence to support that laparotomy promotes tumor progression in CT26 cells with the differentiation of tumor volumes in the laparotomy group and the control group. A higher vessel density was observed in tumor tissues after laparotomy, which indicates an occurrence of enhanced angiogenesis.

Elevated proportions of Tregs usually correlate with unfavorable prognosis in several types of cancer as mentioned above. In the current study, the occurrence of Tregs in laparotomy‐associated cancer tissues was assayed. The increased levels of Foxp3+ and CD4+ cells along with enhanced mRNA expression levels of Foxp3 in the laparotomy group strongly indicate that laparotomy can result in an increase in Tregs population in tumor tissue. Flow cytometry analysis showed that laparotomy also increased the Tregs population in peripheral blood. Chemokines are reported to take part in angiogenesis and immune cells recruitment, and four groups of chemokines were identified of which CXC‐chemokines show the potential to correlate with the progression of malignant tumors (Kakinuma & Hwang, 2006). Among all the chemokines, we investigated the functional contribution of chemokines CXCL1, CCL2, CCL18, CCL22, and CCL28. The mRNA expression levels of target chemokines indicated CCL18 was the only chemokine that was significantly upregulated in laparotomy group, whereas the subsequent IHC analysis showed the protein level of CCL18 was increased as well. Meanwhile, ELISA assay confirmed that CCL18 protein level in the peritoneal cavity was increased, along with the relationship between the Foxp3 level and the CCL18 level in tumor tissues was shown. Both results suggested laparotomy could upregulate the expression of CCL18.

Previous studies have barely focused on revealing the relationship between Tregs and CCL18 in a pathological‐dependent condition. The chemokine receptors CCR6 and CCR7 have been reported to be involved in the presence of Foxp3+ Treg cell in favor of progression of laryngeal squamous cell carcinoma (Chen et al., 2013). A recent study showed that in hypoxia hepatocellular carcinoma cells, CCL28 was demonstrated to be responsible for recruitment of Tregs and eventually resulted in tumor growth in liver cancer (Ren et al., 2016). So far, the mechanism by which the Treg cells migrate to the specific site and promote tumor growth is still not fully revealed for many types of cancer. In our study, we demonstrated that upregulated CCL18 promoted the recruitment of Tregs into tumor tissues. The result showed the knockdown of CCL18 partially destroyed laparotomy‐induced upregulation of Foxp3, whereas CCL18 knockdown significantly inhibited laparotomy‐induced Tregs accumulation in peripheral blood. Moreover, in transwell assay, the downregulation of CCL18 significantly inhibited Tregs migration. In previous studies, CCL18 was demonstrated as a potential regulator in tumor progression and metastasis in which enhanced CCL18 expression level was found in various tumor tissues in presence of several malignancies (Li et al., 2012; Schutyser et al., 2005). In our study, we addressed that the knockdown of CCL18 partially interrupted laparotomy‐induced tumor growth, whereas knockdown of CCL18 also suppressed laparotomy‐induced angiogenesis. In addition, survival analysis of the colon cancer patients showed Tregs in the high percentage group had a low survival rate. Furthermore, a positive relationship was confirmed between Tregs proportion and CCL18 expression.

In conclusion, we demonstrate that laparotomy promotes tumor progression with upregulation of CCL18 observed in laparotomy‐induced tumor tissue. The presence of CCL18 contributes to the subsequent recruitment of Tregs which shows relatively increased population in tumor tissues and peripheral blood after laparotomy. Collectively, our study suggests that CCL18 might be an effective indicator for angiogenesis and tumor growth and a potential target for cancer treatment towards postsurgical recovery.

## CONFLICTS OF INTEREST

The authors declare that they have no conflicts of interest.

## AUTHOR CONTRIBUTIONS

P.X. was responsible for conducting the study, under the supervision of C.M., contributed to the experimental design, did the experiments, analyzed the data, and wrote the paper. All authors read and approved the final manuscript.

## COMPLIANCE WITH ETHICAL STANDARDS

All research and procedures involving human subjects were approved by the ethics committee of Fudan University Shanghai Cancer Center. The procedures in this study were carried out in accordance with the approved guidelines by Fudan University Shanghai Cancer Center. All donors provided a written informed consent for participation.

## Supporting information

Supporting Information Table S1. Primer sequence used in the study
