# Supplementary material for: Surgical trauma‐induced CCL18 promotes recruitment of regulatory T cells and colon cancer progression
Source: J Cell Physiol. 2018 Sep 14;234(4):4608–16. doi: 10.1002/jcp.27245 (PMC6585982; doi:10.1002/jcp.27245)
Supplement: Supplementary file 1 — Supporting Information Table S1. Primer sequence used in the study [file JCP-234-4608-s001.doc]

| *Gene* | Name | Sequence (5’-3’) |
| --- | --- | --- |
| CXCL1    CCL2    CCL18  CCL22    CCL28    Foxp3 | Primer S  Primer A  Primer S  Primer A  Primer S  Primer S  Primer S  Primer A  Primer S  Primer A  Primer S  Primer A | GAATGGTCGCGAGGCTTG  GGACACCTTTTAGCATCTTTTGG  TCAAGAGAGAGGTCTGTGCTGAC  GAGGTGGTTGTGGAAAAGGTAGT  CCAGGTGTCATCCTCCTAACC  CACAGATCTGCCGGCCTCT  TGGTGGCTCTCGTCCTTCTT  GGTGACGGATGTAGTCCTGG  TGAGGTGTCTCATCATGTTTCCG  CTTCCTGCTGGGTTGTTTTTTCC  TTTCACCTATGCCACCCTTATCC  GCTCCCTTCTCGCTCTCCACT |

**Supporting Table S1.** Primer sequence used in the study
